# How can patients with anxiety and/or depression maintain wellbeing following discharge from low-intensity interventions? A qualitative exploration of patient perspectives

**DOI:** 10.1186/s12888-026-08135-5

**Published:** 2026-07-11

**Authors:** Saher Nawaz, Penny Bee, Cintia Faija

**Affiliations:** 1 Division of Nursing, Midwifery and Social Work, School of Health Sciences, Faculty of Biology, Medicine and Health, Manchester Academic Health Science Centre, University of Manchester, Manchester, M13 9PL UK; 2 Department of Primary Care & Mental Health, Institute of Population Health, Faculty of Health and Life Sciences, University of Liverpool, Liverpool, UK

**Keywords:** Anxiety, Depression, Low-intensity CBT, Relapse prevention, Qualitative, NHS talking therapies

## Abstract

**Background:**

Over 500 million people experience anxiety and/or depression globally, underscoring the need for accessible and sustainable psychological support. In England, Talking Therapy (TT) services provide cost-effective, evidence-based low-intensity interventions for mild to moderate anxiety and/or depression. Although over half of patients reach recovery by the end of treatment, relapse rates exceed 50% within one year, highlighting the importance of understanding how recovery is maintained post-discharge. This study examined current practices supporting the maintenance of treatment gains, identified factors that help or hinder sustained recovery, and explored opportunities to strengthen long-term relapse prevention support.

**Methods:**

Semi-structured interviews were conducted with patients (*N* = 23) who met recovery criteria for anxiety and/or depression at the end of low-intensity treatment delivered across four NHS Trusts in Northern England. Data were analysed using the Consolidated Framework for Implementation Research (CFIR) to explore multilevel influences on recovery maintenance.

**Results:**

Findings mapped onto CFIR domains. Within the *intervention* domain, relapse-prevention components were limited and inconsistently delivered, with many patients receiving only brief discussion or written materials. In the *inner setting*, discharge processes and follow-up practices varied, creating gaps in structured maintenance. *Outer-setting* influences such as employment stressors, bereavement, and access barriers hindered recovery. At the level of *individual characteristics*, memory difficulties, self-regulation demands, and reliance on social networks shaped the sustainment of treatment gains and wellbeing. *Process* issues included the absence of planned transitions to community resources, highlighting opportunities to strengthen support pathways. Participants proposed improvements spanning both *clinical maintenance* (e.g., routine follow-up, consolidation of skills) and *personal recovery* (e.g., enhanced social/peer networks and clearer community linkages) to promote long-term wellbeing.

**Conclusion:**

The study highlights the need to strengthen both clinical relapse-prevention practices and broader personal-recovery supports to enhance long-term outcomes following low-intensity psychological treatment. Although situated within NHS TT services, the mechanisms identified—self-regulatory maintenance processes, memory-related barriers, and social-network influences—are internationally relevant and align with global efforts to scale brief psychological interventions. These findings provide transferable insights for improving sustained recovery in diverse mental-health systems.

**Clinical trial number:**

Not applicable.

**Supplementary Information:**

The online version contains supplementary material available at https://doi.org/10.1186/s12888-026-08135-5.

## Introduction

Anxiety and depression are among the most common mental health disorders worldwide [1], affecting over 500 million people [2]. In the United Kingdom (UK), they represent a major public health concern, with 16% of adults reporting moderate to severe depression symptoms [3] and 23.4% experiencing high levels of anxiety [4]. These have wide-reaching consequences, including poor physical health [5, 6], impaired daily functioning [7, 8], reduced quality of life [9], heightened suicide risk [10] and significant economic burden [11, 12]. They also impact the workplace, contributing to poor concentration, sickness absence, and reduced work performance [13–15].

Access to effective mental health support is therefore vital for both individual wellbeing and public health. Demand for psychological and pharmaceutical interventions has risen, with up to 75% of patients expressing a preference for psychotherapy over medication [16–18]. The National Institute for Health and Care Excellence (NICE) recommends psychological treatment as a first-line intervention for anxiety and depression [19, 20]. In England, NHS Talking Therapies (TTad) provides stepped-care, evidence-based treatment in line with NICE guidelines [21]. Step 2 care typically involves low-intensity, cognitive behavioural therapy (CBT)-based interventions, delivered by Psychological Wellbeing Practitioners (PWPs) to patients with mild to moderate symptoms.

In TT services, progress is primarily assessed in terms of clinical recovery, defined by symptom reduction on outcome measures such as the Patient Health Questionnaire-9 (PHQ-9) [22] for depression, the Generalised Anxiety Disorder-7 (GAD-7) questionnaire [23] for anxiety and the Work and Social Adjustment Scale (WSAS) [24] for impairment of functioning. However, research has increasingly highlighted the distinction between clinical recovery and personal recovery [25, 26]. While the former emphasises symptom remission, personal recovery stresses empowerment, hope, identity, and the ability to live a meaningful life despite ongoing difficulties [27–29]. Thus, relapse can be defined in various ways, including clinical, functional, and subjective perspectives. In this study, we adopted a clinical definition, consistent with NHS TTad services, which operationalise recovery and relapse through Routine Outcome Monitoring (ROM) measures. Supporting both forms of recovery requires equipping patients with effective self-management strategies [30–32]. These can include establishing routines, maintaining a healthy lifestyle, and continuing to utilise therapeutic skills after discharge.

The process of sustaining the recovery journey can be conceptualised through models of behaviour change. The Transtheoretical Stages of Change Model [33], for example, describes recovery as a staged, ongoing process rather than a discrete event. While treatment often targets the early stages of change (pre-contemplation through action), the later stages of maintenance and termination are particularly relevant post-treatment, as they emphasise the long-term application of skills learned during therapy [34]. This perspective highlights the importance of relapse prevention strategies in consolidating treatment gains. Relapse prevention is a highly influential framework across behavioural health, with its core concepts heavily shaped by foundational research in substance use disorders [88, 89].

Despite the effectiveness of TT services, where around 50% of patients achieve recovery criteria at discharge [35], relapse remains a significant challenge. Up to 79% of patients experience symptom return within six months of low-intensity treatment [36], and 65.8% relapse within one year [37]. Moreover, 13.7% of patients re-enter services within one to five years, regardless of whether they initially recovered [38]. These findings underline the need to strengthen support beyond discharge to sustain recovery over time.

There is increasing recognition that relapse prevention should be an integral part of treatment [39, 40]. Stand-alone relapse prevention interventions, or those combined with medication, can reduce the likelihood of relapse in depression [41]. However, in TT services, the integration of relapse prevention into routine low-intensity practice remains inconsistent and poorly understood. Research on relapse prevention following guided self-help is particularly sparse [42]. In light of these gaps, the present study investigates the experiences of patients who achieved clinical recovery at discharge from low-intensity TT interventions. It aims to provide an in-depth understanding of current practices that support the maintenance of treatment gains, explore the challenges patients face in sustaining recovery post-discharge, examine the strategies they use to overcome these challenges, and identify opportunities to strengthen relapse prevention within routine practice.

## Methods

This study is part of a larger research project, The CO-IMPROVE project, aiming to improve long-term benefits for patients following discharge from low-intensity treatment for anxiety and/or depression in TT services in the north of England. Ethical approval was granted by the NHS Health Research Authority and Health and Care Research Wales (Reference: 23/NW/0109). Research governance was approved at all participating NHS TT sites.

### Study design

This qualitative study employed semi-structured interviews to explore patients’ experiences following discharge from low-intensity interventions in TT services. The interview schedule (S1 Appendix A) was developed in collaboration between the research team and the Patient and Public Involvement (PPI) advisory group. The interview schedule was informed by (but not limited to) the domains within the Consolidated Framework for Implementation Research (CFIR) [43, 44]. A PPI member piloted the interview before recruitment started. The interview schedule was regularly reviewed and refined during data collection.

### Sample

Participants were eligible if they: (a) were 18 years or older, (b) had started low-intensity treatment with case-level anxiety and/or depression at a participating TT service located in the North of England, (c) met TT recovery criteria in the final session, and (d) were discharged within six months after achieving TT recovery criteria at the time of participation in the study. TT recovery criteria was measured by ‘caseness’. A patient is at ‘caseness’ when their symptom scores exceed the accepted clinical threshold for the relevant measure of their symptoms, i.e., for the GAD-7 ‘caseness’ is indicated by a score of 8 or more and for the PHQ-9 ‘caseness’ is indicated by a score of 10 or more. A person is deemed recovered if their symptoms were considered a clinical case at the start of treatment and not a clinical case at the end [45]. The estimated sample size was 20–25, guided by the principle of theoretical sufficiency, which is informed by both the volume and richness of the data collected [46]. This approach was chosen over the concept of data saturation, which suggests that data collection continues until no new insights are generated indicating that a point of complete informational redundancy can be achieved [47]. Theoretical sufficiency, in contrast, emphasises gathering enough rich and varied data to develop well-supported interpretations and theoretical insights [47, 48]. This approach acknowledges the iterative and interpretative nature of qualitative interviews, whereby engagement with new participants and refinement of interview questions can continue to generate valuable insights. This approach ensures that the data support well-grounded interpretations and theoretical insights [48] while allowing recruitment to continue until sufficient depth has been achieved to address the research question meaningfully. In practice, sufficiency was judged through ongoing analysis during data collection, ensuring that the data provided adequate depth across participants’ experiences to meaningfully answer the research questions.

### Data collection and procedure

This study was based in northern England. Four NHS Trusts across TT services supported recruitment. Eligible patients were identified by NHS staff and were invited by them to participate. The *recruitment approaches varied across sites*,* reflecting local capacity*,* capability*,* and population characteristics.* At one site, all eligible individuals were contacted directly; at a second site, recruitment focused on participants from ethnic minority backgrounds to enhance diversity. The remaining two sites used a combination of practitioner referral and support from Research and Development teams.

Recruitment for this study commenced on 18th May 2023 and concluded on 9th April 2024.

Invited participants received an invitation pack via email or post, comprising an information sheet, a consent-to-contact form, an invitation letter and a recruitment advert. Interested participants approached the research team directly or provided consent to NHS staff to share their details with the research team, who then approached them. Those who agreed to participate provided informed consent online (via Qualtrics) before participating. Following consent, participants completed an online demographics form including background details (e.g., age, gender, ethnicity) and the ROM measures completed during treatment (i.e., PHQ-9, GAD-7, WSAS). The use of the ROM questionnaires enabled an effective measurement of patients’ relapse status at the time of data collection. *ROM scores following discharge were used as a clinical frame to identify individuals with lived experience of relapse and those who remained well following discharge*,* and to better understand factors that may have helped or hindered these experiences. ROM data following discharge were used to sensitise and prompt interview questions.* Patients whose scores fell within the thresholds designated by the TT services, which indicate potential caseness, were classified as not being in a state of recovery, but rather experiencing a relapse. In this study, we define relapse according to established definitions and current clinical guidelines as the re-emergence of symptoms occurring within six months following the achievement of treatment recovery, but prior to the maintenance of long-term gains. Additionally, participants received an optional questionnaire (2 Appendix B) to provide written qualitative details of their experiences during and after treatment before the interview, related to their recovery journey. This questionnaire comprises 6 questions and was developed in collaboration with the PPI advisory group, who deemed this information valuable for understanding and contextualising the numerical scores from the measures and prompting interview questions. In this optional questionnaire, participants were invited to discuss several critical aspects of their treatment experience including: the benefits derived from the treatment, the plans/strategies (if any) developed with their practitioner to support ongoing recovery, the challenges encountered after completing treatment, along with any support received during this period and their recommendations/suggestions for improving care to assist themselves and others better following treatment.

All interviews were conducted by SN. The semi-structured interview schedule was designed to guide discussion across three core areas aligned with the study aims: (1) views and experiences of relapse prevention and end-of-treatment planning; (2) barriers and facilitators influencing the maintenance of treatment gains and relapse (including resources used, and factors that helped or hindered); and (3) identifying necessary post-treatment care, support, and resources. Examples of questions included: What were the recommendations/discussions (if any) you had with your practitioner to maintain recovery and prevent relapse?, What were the challenges you have faced to maintain recovery following treatment?, How were you supported following the completion of your treatment?, and What could be done differently to help people following treatment? The interview schedule was developed through a systematic and iterative process. The initial draft was informed by key literature on relapse prevention and behavioural maintenance models (e.g., the Behaviour Change Wheel; BCW). The draft was refined by our Patient and Public Involvement and Engagement (PPIE) advisory group to ensure accessible, non-judgmental language and topic prioritisation based on patient needs. Following this refinement, the schedule was pilot-tested with a PPIE member to confirm clarity, flow, and timing, with only minor adjustments made before data collection.

Interviews were recorded online via Microsoft Teams or Zoom. Interviews were transcribed verbatim by SN or a university-approved independent transcription service, checked for accuracy, and anonymised to ensure confidentiality. All data were stored securely in compliance with university guidelines.

### Data analysis

Data analysis was guided by the CFIR using a framework analysis approach [49, 50]. This method enabled structured, deductive coding against a priori CFIR domains and constructs while remaining open to inductively capturing participants’ experiences that extended beyond the framework. The study is informed by a social constructionist epistemology [51], which recognises that knowledge is co-constructed in social contexts rather than passively received. Combining framework analysis with this epistemological stance allowed for a systematic examination of implementation factors while valuing the interpretive contributions of both participants and researchers.

The CFIR is widely used in and beyond implementation science [44, 52–54], providing a comprehensive framework of 39 constructs across five domains: (1) Intervention characteristics, (2) outer setting, (3) inner setting, (4) characteristics of individuals, and (5) process. In this study, it served as an organising framework for identifying factors influencing current provision and opportunities for relapse prevention within TT services. Participants’ narratives were examined in relation to CFIR to organise and interpret accounts of contextual, interpersonal, and system-level influences on maintenance and relapse prevention.

The analysis process involved several stages, and a worked example is provided in S4 Appendix C. First, researchers familiarised themselves with the data through multiple readings of the transcripts. Second, data were coded deductively against the CFIR framework. Where participant experiences did not map onto existing constructs, inductive codes were generated. Inductive coding was applied selectively to sections of the data that did not fit clearly within existing CFIR constructs. These inductive codes were then examined to determine whether they extended, nuanced, or refined CFIR constructs in ways relevant to the present study. Where appropriate, CFIR construct definitions were tailored to reflect these inductive insights. Third, data were charted and organised into matrices to facilitate comparison both within and across cases. Fourth, preliminary findings were reviewed and refined through discussion with the wider team and the Patient and Public Involvement (PPI) group, to enhance reflexivity, interpretation, relevance, and credibility of the findings. While the analysis began with the full set of CFIR domains and constructs, the coding process revealed that some constructs within domains lacked empirical relevance in our data. It is important to note that no domains or constructs were removed a priori. Rather, constructs were only omitted from the final report if no relevant data mapped onto them during the analysis. Conversely, where participant experiences yielded unique insights that did not align with existing CFIR constructs, inductive codes were generated and subsequently organised as new constructs within the most appropriate CFIR domain to ensure comprehensive coverage of the contextual factors.

All transcripts were coded by SN, with CF providing regular supervision. This included reviewing coding decisions and ensuring consistency in mapping data onto CFIR domains and constructs, and incorporating inductive insights. QSR International’s NVivo-12 qualitative software was used to manage and organise the data [55].

### Quality

This study adhered to the standards outlined in the reporting of qualitative research (SRQR) and the consolidated criteria for reporting qualitative research (COREQ) to enhance transparency and ensure the quality, validity, and reliability of the research conducted [56]. Several strategies were employed to ensure the trustworthiness. Reflexivity was maintained throughout, with the first author (SN) documenting reflections on their positionality and potential influence on data collection and interpretation. Coding was conducted systematically using NVivo 12, with all transcripts reviewed multiple times. Initial coding by SN was discussed iteratively with CF, an experienced qualitative researcher, to ensure alignment with CFIR constructs and consistency in interpreting inductively identified data. Preliminary findings were also shared with the PPI group to confirm that interpretations accurately reflected participants’ experiences. An audit trail of coding decisions and analytic memos was maintained to support transparency and replicability.

### Reflexivity and positionality

All interviews and initial coding were conducted by SN, who has no clinical experience as a PWP and no prior exposure to TT services. This outsider perspective provided a fresh lens on patients’ experiences and enabled open exploration of service practices and relapse prevention. Rather than treating positionality solely as a potential bias, we recognised SN’s standpoint as an analytic resource that actively shaped both data collection and early interpretation.

SN was supported by CF, who has clinical experience and extensive qualitative research expertise in NHS TTad contexts, though not practising clinically within these services. This strengthened interpretation by grounding analysis in practical service realities, while we scrutinised this stance to ensure pragmatic constraints were not accepted as fixed and did not limit interpretations by feasibility. We mitigated bias through iterative team discussion, deviant-case analysis, transparent construct placement, and use of quotations. CF’s guidance ensured that coding and interpretation were rigorous, and aligned with the CFIR framework. Supervision involved a detailed review of data mapped to CFIR domains and constructs, discussion of alternative interpretations, and reflexive consideration of construct placement and definition. Illustrative quotations were reviewed collaboratively to support consistency and transparency.

Consistent with a social constructionist epistemology [57], we understood knowledge as co-constructed between researchers and participants, rather than passively received. Iterative discussions between SN and CF functioned not simply as reliability checks, but as a reflexive space in which differing standpoints were deliberately used to refine and question emerging interpretations. This process strengthened analytic rigour by making the influence of researcher positioning visible and accountable, ensuring analytic decisions were transparent, interpretations were grounded in participants’ experiences, and that the team’s complementary perspectives were harnessed as a strength.

## Results

Across four NHS trusts, 53 participants expressed interest. Of these, 25 individuals provided informed consent, and 23 were interviewed. The two participants who consented but did not proceed with the interview withdrew prior to data collection commencing, citing employment difficulties and health issues, respectively. Those who expressed initial interest did not respond to subsequent contact. Of the 23 participants who took part, 11 completed the optional questionnaire.

A total of 14 female and nine male patients aged between 27 and 83 years (M = 54.5, SD = 16.1) participated in this study, with the majority identifying as White British (n = 18). The ethnicity of the remaining five participants included two individuals identifying as white and other, one as white and black Caribbean, one as Asian (Pakistani), and one as ‘other’ ethnic group. Additionally, 14 patients did not identify as having a disability, while nine reported having one. Regarding the source of referral, 19 patients self-referred, three were referred by a GP, and one did not specify the referral route. Concerning relationship status, 11 patients were married, five were cohabiting, three were single, two were divorced, one was non-cohabiting and one other. Most patients identified themselves as Christian (*n* = 11), four described themselves as agnostic, four as atheist, two as Muslim, one as having a different religious background, and one as having no religion. Most participants in the study were employed full-time (*n* = 10) and held educational qualifications at least at degree level (first/ordinary) or higher (*n* = 13). In terms of treatment modalities, nine participants received treatment by telephone, eight underwent face-to-face treatment, and three received a combination of telephone and face-to-face treatment. Additionally, one participant utilised online CBT, one engaged in video treatment, and one did not specify the treatment format. Additional details, including individual measure scores, are presented in Table 1.

**Table 1:** Participant demographics, service, time since discharge, and routine outcome measure (ROM) scores (low, mild, moderate, severe)

ID	Service	Age (Years)	Time since discharge (Months, Days)	PHQ-9 score (Start / Discharge / Prior Interview)	GAD-7 score (Start / Discharge / Prior Interview)	WSAS score (Start / Discharge / Prior Interview)
PAT01	B	62	2, 9	12 / 6 / 3 Moderate → Mild → Mild	11 / 6 / 1 Moderate → Mild → Mild	21 / n/a / 19 Severe → n/a → moderate
PAT02	A	58	2, 1	13 / 6 / 7 Moderate → Mild → Mild	6 / 2 / 5 Mild → Minimal → Mild	26 / 11 / 22 Severe → Moderate → Severe
PAT03	A	71	1, 10	27 / 1 / 0 Severe → Minimal → Minimal	14 / 0 / 0 Moderate → Minimal → Minimal	21 / 0 / 0 Severe → Low → Low
PAT04	B	54	3, 6	11 / 7 / 8 Moderate → Mild → Mild	9 / 7 / 6 Mild → Mild → Mild	13 / 6 / 7 Moderate → Low → Low
PAT05	B	68	3, 8	15 / 6 / 4 Moderately Severe → Mild → Minimal	14 / 6 / 9 Moderate → Mild → Mild	15 / 7 / 7 Moderate → Low → Low
PAT07	B	61	3, 8	7 / 6 / 4 Mild → Mild → Minimal	8 / 6 / 5 Mild → Mild →Mild	15 / 12 / 7 Moderate → Moderate → Low
PAT10	A	27	3, 8	10 / 2 / 4 Moderate → Minimal → Minimal	15 / 4 / 2 Severe → minimal → minimal	21 / 2 / 4 Severe → Low → Low
PAT11	B	83	3, 5	16 / 8 / 0 Moderately Severe → Mild → Minimal	9 / 2 / 0 Mild →Minimal → Minimal	15 / 5 / 0 Moderate → Low → Low
PAT14	A	27	2, 24	5 / 3 / 2 Mild → Minimal → Minimal	14 / 4 / 5 Moderate →Minimal → Mild	2 / 8 / 8 Low → Low → Low
PAT16	B	34	3, 16	11 / 6 / 7 Moderate → Mild → Mild	10 / 4 / 5 Moderate → Minimal → Mild	10 / 2 / 10 Moderate → Low → Moderate
PAT19	C	39	4, 3	7 / 4 / 2 Mild → Minimal → Minimal	16 / 2 /3 Severe → Minimal → Minimal	20 / 0 / 0 Severe → Low → Low
PAT20	B	51	3, 26	20 / 0 / 3 Severe → Minimal → Minimal	17 / 2 / 2 Severe → Minimal → Minimal	18 / 1 / 3 Moderate → Low → Low
PAT21	C	65	5, 0	19 / 6 / 10 Moderately Severe → Mild → Moderate	10 / 6 / 4 Moderate → Mild → Minimal	20 / 12 / 10 Severe →Moderate → Moderate
PAT22	A	61	1, 21	9 / 5 / 7 Mild → Mild → Mild	12 / 4 / 3 Moderate → Minimal → Minimal	21 / 6 / 8 Severe → Low → Low
PAT23	A	33	1, 0	23 / 3 / 8 Severe → Minimal → Mild	21 / 3 / 7 Severe → Minimal → Mild	34 / 9 / 14 Severe → Low → Moderate
PAT25	B	58	2, 30	12 / 2 / 3 Moderate → Minimal → Minimal	21 / 1 / 3 Severe → Minimal →Minima	13 / 1 / 5 Moderate → Low → Low
PAT26	B	71	5, 10	7 / 3 / 3 Mild → Minimal → Minimal	8 / 5 / 4 Mild → Mild → Minimal	0 / 1 / 0 Low → Low → Low
PAT27	B	66	3, 13	17 / 6 / 5 Moderately severe → Mild → Mild	17 / 7 / 10 Severe → Mild → Moderate	11 / 6 / 5 Moderate → Low → Low
PAT28	B	70	4, 9	8 / 4 / 4 Mild → Minimal → Minimal	12 / 3 / 5 Moderate → Minimal → Mild	6 / 3 / 0 Low → Low → Low
PAT29	C	30	1, 25	10 / 2 / 3 Moderate → Minimal → Minimal	14 / 3 / 4 Moderate → Minimal → Minimal	14 / 4 / 6 Moderate → Low → Low
PAT32	C	59	3, 13	15 / 7 /12 Moderately severe → Mild → Moderate	20 / 6 / 8 Severe → mild → Mild	16 / 10 / 15 Moderate → Moderate →Moderate
PAT33	C	65	5, 21	4 / 0 / 1 Minimal → Minimal → Minimal	13 / 1 / 2 Moderate → Minimal → Minimal	6 / 0 / 2 Low → Low → Low
PAT34	C	35	1, 11	15 / 1 / 12 Moderately Severe → minimal → Moderate	13 / 3 / 9 Moderate → Minimal → Mild	24 / 2 / 15 Severe → Low → Moderate

Note. This table shows patient scores on TT metrics at different time points, illustrating changes in symptom severity to provide an indication of each patients’ clinical state at the point of the interview. Scores are presented as raw values and as categorical labels with thresholds defined individually for each measure. The categorical labels for PHQ-9 scores are as follows: 0–4 = Minimal, 5–9 = Mild, 10–14 = Moderate, 15–19 = Moderately Severe, and 20–27 = Severe depression. The categorical labels of GAD-7 scores are as follows: 0–4 = Minimal, 5–9 = mild, 10–14 = moderate and 15–21 = severe anxiety. The categorical labels of WSAS scores are as follows: 0–9 = low impairment, 10–19 = moderate impairment, 20–40 = severe impairment. The categorical labels provide a quick indication of severity, while raw scores offer detailed information for further interpretation of recovery trajectories. n/a = Not Available

Findings are organised using the CFIR to reflect multilevel influences on post-discharge maintenance and relapse prevention across its five domains: (1) Intervention Characteristics, (2) Outer Setting, (3) Inner Setting, (4) Characteristics of Individuals, and (5) Process. The analysis combined deductive coding with inductively derived subconstructs to reflect aspects of recovery that extended beyond existing CFIR categories, including 24 original CFIR constructs and 9 emerging inductively.

Across domains, participants described two interconnected but distinct needs influencing long-term recovery: **(1) Core clinical maintenance practices**, such as structured discharge preparation, consolidation of therapeutic skills, and planned follow-up; and **(2) Broader personal-recovery supports**, including social networks, access to community resources, and support for wellbeing beyond symptom reduction.

**Fig. 1:**
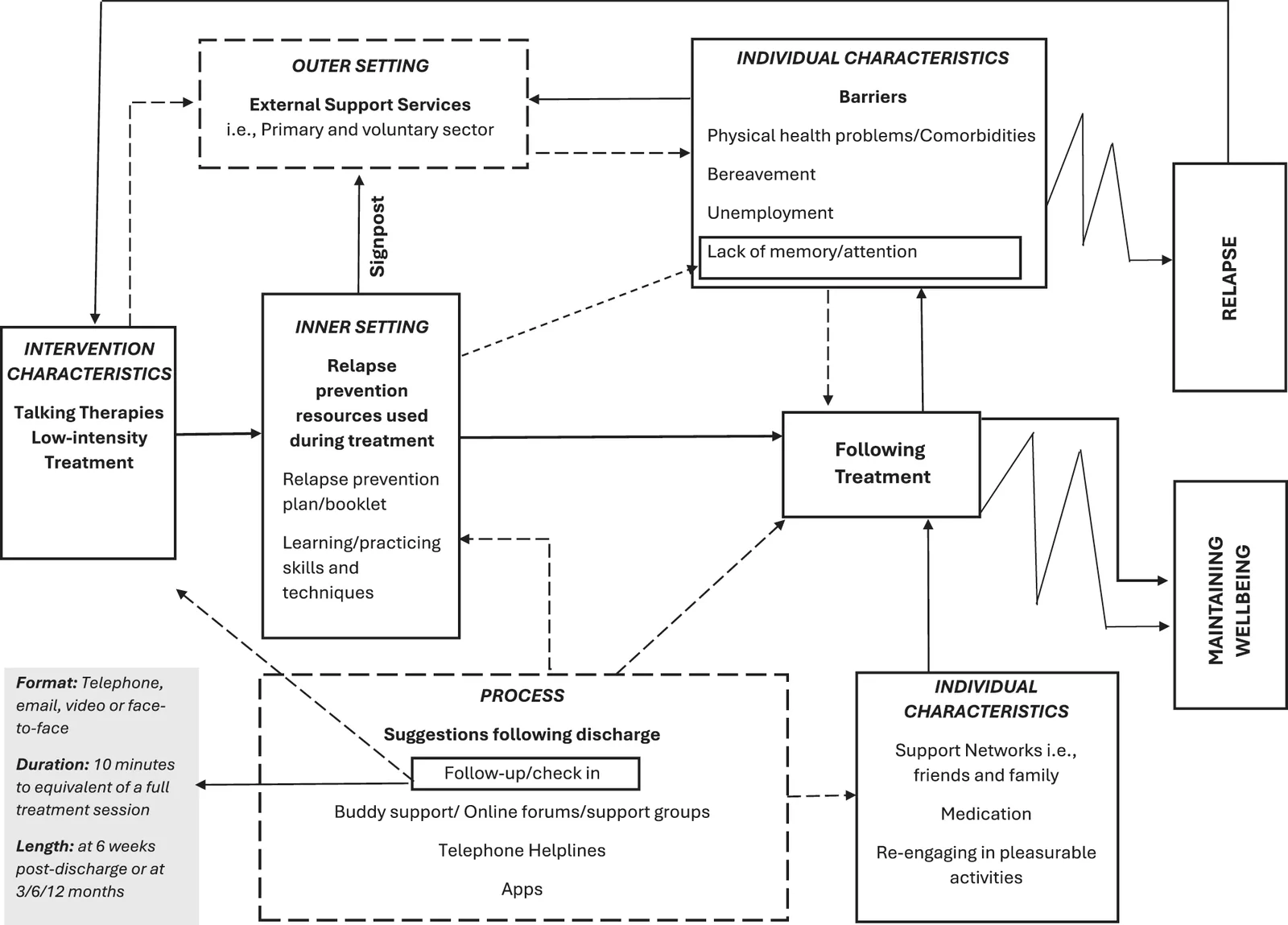
A diagram highlighting the recovery journey as depicted through participants in the present study, the relevant CFIR domains in capitals and the links between each stage/process. The dashed lines (----) represent areas which do not exist or are not well-established and need improvement. The lines going up and down represent the challenges during the recovery journey

Figure 1 illustrates the multifaceted nature of the recovery journey reported by participants, ranging from stable self-management to fluctuating progress or relapse, shaped by support systems available during and after recovery. These trajectories highlight the interplay between CFIR domains and the wider contextual factors influencing recovery pathways. Detailed findings for each CFIR domain are presented below, with illustrative quotations and construct summaries provided in Table 2 and Supplementary File 3.

**Table 2:** Summary of CFIR constructs, definitions and illustrative quotes

Domain	Constructs - Subconstructs	Definition	Illustrative Quotes
Intervention Characteristics	Relative Advantage – Treatment*	Benefits experienced from treatment, including improved daily functioning and social engagement	“So, part of the CBT that was most useful was seeing things in proportion. I live on my own and when you’re not feeling very well, … you tend to catastrophise things, and the CBT taught me not to do that…” PAT02
	Relative advantage – Post-Treatment Care*	Potential benefits of continued support post-treatment to sustain recovery and identify early signs of potential relapse	“So, I think having, like, check-ins … for, like sort of, more vulnerable people then that might be beneficial.” PAT10
	Adaptability	Tailoring of treatment to individual needs (e.g., flexible scheduling, customised modules)	“I really liked how I was able to have an appointment at half eight, which massively helped…” PAT34
	Complexity	Difficulties with completing questionnaires, including limited time and response options	“I find it a bit difficult sometimes to quantify how you feel, … it’s not always easy to quantify in a questionnaire… it doesn’t really translate always, accurately.” PAT23
	Design Quality & Packaging	Perceptions of diverse treatment modalities and formats for materials	“As I’ve got older concentrating on written material is probably more difficult for me than it used to be.” PAT27
	Intervention Context* – Techniques*	Techniques and skills learned during treatment to aid recovery	“She used some analogy with a courtroom and putting negative thoughts on trial …I thought that was good. And problem solving through stuff, which, yeah, I thought that was helpful. And identify triggers as well.” PAT20
Outer Setting	Cosmopolitanism	Links between TT services and external services (GPs, other health/financial support)	“All I’ve had since treatment finished, was a phone call off my doctor’s asking how I was doing. Basically, that’s it.” PAT04
Inner Setting	Structural Characteristics	Speed and accessibility of the service	“I was very lucky actually I got mine fairly rapidly … I was spoken to within a couple of weeks. Whereas I know some of my patients are waiting months.” PAT20
	Culture	Organisations’ values, especially the significance of shared decision-making	“She gave me the option really, she said, now we could continue you this if you like, or if you wish we can just stop it there. It was four sessions, and I felt happy enough stopping it.” PAT11
	Implementation Climate - Tension for Change	Need for post-treatment support to prevent relapse	“It’s like with anything, you can’t just…people to go through what they’ve been through and then just, that’s it, cold turkey, off you go. Because there’ll be so many people that relapse, because there isn’t easily available care for the person to speak to…” PAT16
	Implementation Climate - Relative Priority	Importance of post-treatment care varies by personal experience	“ I think it’s very important because personally, I don’t consider myself cured from anxiety and I don’t think I will be, so there are always ups and downs. … but it’s easier to prevent recurrence with a more regular tool that they can use.” PAT19
	Implementation Climate - Goals and Feedback	Treatment aligned with patient goals	“ I went there for like guidance and I felt like I got good guidance because they gave me tools that I didn’t have.” PAT25
	Readiness for implementation -Available Resources – During/After Treatment*	Resources provided during and after treatment to support recovery	“ …it’s there and I can access this for a year after the start of the treatment… I’ve downloaded more of the meditations and those are on my phone now. So it’s nice to be able to go back and do that.” PAT02
	Readiness for implementation - Access to Knowledge – During/After Treatment*	Ease of and difficulties in accessing information and support	“They do point you in directions of different services that are available…” PAT25
Characteristics of Individual	Knowledge & Beliefs about intervention – knowledge of relapse prevention* and beliefs of relapse prevention*	Attitudes, values and understanding of relapse prevention and post-treatment care	“It’s a bit more complicated than that. There isn’t an immediate cure. I think it’s one of the things, especially with mental health, that you have to keep at.” PAT02
	Self-Efficacy	Confidence to continue recovery independently	“I have taken away a lot from the last session as well. As in right, although I’m not getting face to face, this doesn’t mean I’ve got to stop doing what I’ve been doing.” PAT04
	Stage of Change	Stage of recovery: action (treatment), maintenance (remaining in recovery), relapse (experiencing a relapse following treatment)	“I realised in early July that I was going downhill and sought treatment immediately.” PAT11 (Action stage)”
	Other Personal Attributes	Motivation and willingness to share learning with others	“ …my journey feels more that I don’t kind of need a lot of support, but I think it’s my time to give and help. I’ve done it for 30 years in a job and I want to do it for a wider society, yeah, that’s where I’m at.” PAT25
	Meaning of Recovery*	What recovery means to patients	“Day by day, I think. It’s taking a day at a time.” PAT07
Process	Planning	Patients’ suggestions for post-treatment care	“Maybe a group of people that have undergone CBT that might want to talk about their experience.” PAT002
	Engaging – Formally appointed internal implementation leaders/ Patients*/Professionals*/External Agents	Individuals involved in delivery and support of interventions	“It could come from community. It could come from family and friends … So, you can talk things over with professionals. But talking over with friends, work colleagues, lots of different people can provide support.” PAT020
	Execution	Carrying out intervention plans	“ … online would be the cheapest option, I would think, for people to be in touch with people.” PAT021
	Reflecting & evaluating	Feedback about the progress and quality of intervention	“ Well, at first, I thought it was a waste of time and then at the end I thought it was very good.” PAT011

Note. * Represents newly developed constructs within the CFIR. N/A = Not available

### Intervention characteristics

The intervention characteristics domain captures participants’ accounts of the treatment they received and what they found helpful in relation to recovery. While the CFIR analysis focused on predefined aspects of intervention design and delivery, discussions within the research team considered how participants’ reflections extended beyond technical features to encompass relational and experiential dimensions of care.

Participants expressed that the intervention was ‘*tailored to [their] experience*’(PAT02) and needs. Participants frequently elaborated on what “tailoring” meant in practice when prompted for clarification, suggesting that aspects of personalisation are not taken for granted within routine service delivery. This included providing access to all relevant modules for those using online guided self-help treatment and the flexibility of adjusting the timing of treatment sessions to accommodate the participants’ schedule, ensuring they can ‘*keep to the appointment*’ (PAT34). One participant highlighted the value of the treatment refinement process achieved through practising different techniques to find what was most suitable or not and why:

*I think it was more just going over things that worked for me*,* and I think that we’d tried over the past seven or eight months*,* what didn’t work*,* why didn’t it work*,* those kind of things really.* (PAT16)

The data uncovered several strategies that participants learned and utilised during treatment and continue to use following treatment. Six participants found ‘worry time’ (allocating time to worry about things) to be valuable in overcoming their excessive worrying, whilst five participants emphasised the benefits of journalling or maintaining a worry diary (recording worries) to manage their symptoms. Three participants discussed the practicality of categorising worries as hypothetical or practical to manage their symptoms of anxiety and/or depression. Additionally, five participants cited the usefulness of breathing exercises and relaxation techniques when symptoms of anxiety resurface. Other techniques mentioned included the ‘courtroom technique’, which involves ‘*putting negative thoughts on trial’* (PAT20) and determining *‘is it real?*’ (PAT05).

Participants also stressed the benefits of undergoing treatment. The core element of ‘*just speaking to someone that [they] didn’t know’* (PAT10) was advantageous due to the absence of preconceptions of the participant by the therapist. The therapist’s practice of highlighting the achievements the patient made during treatment was effective. Furthermore, the ability to complete daily tasks, establish a regular routine, and notably, increased social confidence were listed as benefits of the treatment for many participants. The improvement in daily routine was reassuring, instilling a sense of confidence, and demonstrating that *‘what [they] are doing is working*,* even after therapy’* (PAT10):

*Probably just the ability to talk about it. I think that’s the biggest thing… And often*,* with it being a third party*,* I don’t know why*,* it’s just that little bit easier initially*,* to kind of*,* you end up just blurting it out*,* in comparison to maybe loved ones and things like that.* (PAT16)

All participants acknowledged the value of the treatment and reflected on the potential benefits of implementing and enhancing support following treatment to sustain recovery. Data also revealed that initiatives supporting progress following treatment could ‘*help identify people who were perhaps at a risk further down the line’* (PAT27), which would help to *‘stop people having to access treatment so often’* (PAT05) and empower individuals to continue utilising the techniques over time. Further benefits included reducing waiting lists, and providing valuable opportunities for therapists to monitor long-term effectiveness.

Participants expressed concerns that questionnaires may inaccurately capture a fleeting emotional state rather than the overall experience over a specified period due to the dynamic nature of their mental health. For instance, data highlighted how the questionnaires’ scale posed challenges:

*The answer [was] a little bit more nuanced*,* and [they] had to go with either option*,* which is not entirely a hundred per cent correct* (PAT23)

Additionally, one participant expressed that a question about suicidal thoughts in a questionnaire negatively influenced their self-perception and recovery progress:

*…should I be feeling suicidal? … So the constant mention of killing yourself and harming yourself and suicide wasn’t really in my case beneficial.* (PAT07)

Although this data mapped onto CFIR constructs related to intervention design and evaluation processes, analytic discussions considered whether framing these accounts solely as design limitations risked underplaying participants’ broader critique of standardised measurement in fluctuating mental health contexts.

Participants underscored the value of face-to-face appointments emphasising how a different environment increased comfort, allowing for honest discussion about the problem alongside the ability to ‘*convey… body language’* (PAT23). Conversely, some patients expressed a preference for telephone appointments as thoughts and feelings could be better conveyed as they ‘*didn’t have any preconceived ideas’* (PAT07). In the context of material provided during sessions, such as worksheets and other resources, opinions diverged. Some patients perceived written material as more accessible whereas others found electronic copies more practical as they could ‘*dip in and dip out’* (PAT20) of the information whenever needed.-.

### Outer setting

Data for this domain were limited and primarily related to cosmopolitanism, informed by five participants. This highlights the lack of networks between TT services and external services, specifically with general practice and employment services, where communication was often through a letter. Often, the signposting to external service providers was to allow patients to address topics outside of the remit of mental health, mainly focussing on physical health or financial concerns:

*So*,* she [Therapist] wrote a letter to my GP and I’ve been referred to the pain clinic.* (PAT02)

Participants’ limited reference to inter-organisational networks may reflect their relative invisibility to service users rather than their absence in practice, an interpretation considered during team discussions.

### Inner setting

This domain details participants’ perceptions of TT services shaped by their experiences including the core values of the service, issues that arose during treatment and the resources and knowledge provided to aid their recovery journey (during and after treatment).

Data highlighted the collaborative patient-centred care nature of treatment within TT services evidenced by informed patient choice in the decision-making process between the therapist and the patient, including ‘*mutual decision[s]*’ (PAT28) on treatment format (telephone, online or face-to-face) and agreement for discharge. However, one patient felt that more sessions were necessary, despite the therapist considering them recovered:

*So*,* the therapist said*,* I think you’re good enough to be discharged… it would have been better if I’d have had some warning so I could have put together some thoughts on that. (PAT29)*

While many accounts reflected collaborative decision-making, analytic discussions considered how this framing may align with core service values and documentation practices, and whether participants’ narratives simultaneously revealed more asymmetrical dynamics, particularly around discharge decisions. The team discussed how participants’ accounts also reflected tensions between clinical judgement and patient preparedness, highlighting relational aspects that extend beyond structural categorisation.

One participant highlighted that the end of treatment was several months later than anticipated, and with someone whom they had not built a rapport with due to therapist illness:

*It would have been nice and probably beneficial to have had the continuity. I think it was about a two- or three-month break*,* so I’d kind of forgotten an awful lot.* (PAT07)

Participants emphasised the significance of care following treatment beyond the actual treatment phase, highlighting its prioritisation. This was underscored by the sentiment that “*there’s nothing at the minute*” (PAT16) supporting recovery after discharge. While initially interpreted as a resource gap within the Inner Setting, team discussions explored whether this perception reflected the absence of structured follow-up or the limited visibility of existing pathways to patients at the point of discharge. The perception of the need for care following treatment is heavily influenced by individual experiences post-treatment. Some did not consider post-treatment care as vital, as they have “*managed without it*” (PAT05), but acknowledged the potential benefits. Others regarded support following treatment as crucial, drawing comparisons to physical health approaches:

*You would monitor someone who you’ve prescribed medication*,* but there’s no monitoring for someone who’s been given CBT. (PAT25)*

Participants reported having contact details to seek additional support following treatment, including those of the TT service and crisis services such as Samaritans:

*I was told I could self-refer and everything. Then I got the discharge letter and details of other things that I might like to consider becoming involved in.* (PAT21)

However, data highlighted participants *‘would have liked additional information’* (PAT23) following treatment as they ‘*don’t really know much about different support [they] could get afterwards’* (PAT27). Participants often had to rely upon their own resourcefulness and proactively seek information about support available in their locality through online sources (e.g., internet, YouTube, library or self-help literature). This emphasis on individual initiative prompted reflexive consideration of how responsibility for sustaining recovery may implicitly shift from service to patient once formal treatment ends.

Data revealed resources participants used during treatment including a ‘*booklet for relapse’* (PAT20), *‘leaflets about if [they] relapse’* (PAT33), ‘*link to different NHS websites’* (PAT26), *‘handouts about anxiety issues and cognitive behavioural therapy’* (PAT27), and other forms of paperwork completed during sessions. However, additional resources following treatment were scarce with contact after treatment being a *‘discharge letter’* (PAT21). Participants consistently highlighted the advice by therapists to ‘*refer to the paperwork’* (PAT05) received in treatment to support recovery after treatment.

### Characteristics of individuals

This domain describes how participants’ knowledge, beliefs, capabilities, and personal resources shaped their ability to sustain recovery following discharge. Individual-level factors were closely interwoven with service processes and broader contextual influences, together determining the ease or difficulty of maintaining gains over time.

Many participants were uncertain about what to expect after discharge, particularly what support would remain available or how to re-access the service if difficulties re-emerged: ‘*don’t know what is supposed to happen’* (PAT27);

*I didn’t get into what’s the process (PAT28)*

Although several framed ongoing progress as primarily as *‘down to the individual’* (PAT03), they simultaneously emphasised the importance of knowing a ‘*lifeline*’ (PAT11), ‘*lifejacket’* (PAT26) or ‘*safety net’* (PAT28) existed if needed. These accounts illustrate a tension between autonomy and reassurance. While participants wished to take ownership of their recovery, unclear post-treatment roles and expectations appeared to erode confidence. The desire for a “safety net” reflects not dependence but a need for containment and reassurance during vulnerable periods.

Some participants conceptualised their difficulties as persistent: ‘*it’ll always be there*’ (PAT16); *lurking about in the background’* (PAT25), prompting two participants (PAT23 and PAT26) to question whether *‘prevention [is] better than cure [treatment]’* (PAT026). These narratives framed recovery as a process of long-term management rather than complete resolution. We interpret this as lay models of maintenance, in which relapse prevention is understood as early noticing and timely adjustment rather than a discrete, one-off intervention.

Recovery was commonly described as a personal and non-linear journey characterised by gradual improvement, a ‘*gentle return to normality*’ (PAT01), feeling *‘a lot better*’ (PAT14), or being able to ’*cope with everything’* (PAT32). Fluctuations were expected: ’*It’s a marathon*,* not a sprint*’ (PAT25); ‘*a pendulum’* (PAT26). We interpreted these metaphors as a way of conveying an intuitive understanding of recovery as cyclical self-regulation. Participants anticipated ups and downs and valued forms of support—formal or informal—that helped them translate therapeutic strategies into everyday habits.

Participants identified several contextual pressures that disrupted progress, including work stress, physical ill-health, and bereavement. Practical factors also played a role; for some, accessing help remained “*quite a trek*” (PAT21). Cognitive barriers emerged too, particularly skill drift during periods of relative stability - “*When you don’t really feel like you need it*,* you don’t use it*” (PAT23). Among the five participants who relapsed, triggers included employment or financial stress, personal or family ill-health, and bereavement. Time-to-relapse ranged from 1 to 5 months, highlighting the unpredictability of risk windows. These patterns indicate that contextual stressors can trigger temporary reductions in capacity, which may be compounded by memory-related barriers such as skill decay. This underscores the potential value of light-touch prompts and anticipatory planning to help keep techniques accessible without requiring full re-engagement with therapy.

Despite these challenges, many participants described feeling increasingly confident in applying strategies and recovering more quickly from setbacks, e.g. being able to “*brush it off better*” (PAT16) or feeling “*confident now that I can go and do it on my own*” (PAT14). Therapist feedback highlighting “*there’s been a very big improvement*” (PAT11) and social validation “*my brother said*,* the change in you in three months is unbelievable*” (PAT03) further reinforced progress. These reflections point to the development of self-efficacy, strengthened by both therapeutic and social reinforcement. Confidence operated both as a result of practice and as a protective factor that helped prevent difficulties from escalating.

Family, friends, and peers were described as central sources of ongoing support: “*My wife has been a tremendous support*” (PAT11); “*talking to other people…helps maintain recovery*” (PAT25). Engaging in valued activities (e.g., watching rugby, exercising, spending time outdoors, meditation) offered stability and emotional regulation. Medication also supported some participants in maintaining gains across changing circumstances. These accounts highlight relational and activity-based pathways to maintenance. Social connection and meaningful routines act as buffers to stress, prompt the use of therapeutic techniques, and align with broader personal-recovery goals of living well rather than simply controlling symptoms.

Participants did not suggest changes to the content of the final treatment session but emphasised the importance of ‘*ongoing support’* (PAT34) to help embed skills into everyday life after treatment concludes. We interpret this as a preference for continuity rather than redesign, a desire for light-touch supports that bridge the gap between structured therapy and daily living.

### Process

This domain captures participants’ suggestions for how post-treatment processes could better support long-term recovery. Across the sample, participants articulated a wide range of ideas, indicating both unmet needs and clear opportunities for strengthening maintenance pathways. The most commonly proposed form of post-treatment support was a follow-up or check-in (*n* = 17). Participants varied in their preferences for format (telephone, video call, email or face-to-face), and in timing (ranging from 6 weeks to one year after discharge). The length of contact also varied in suggestions: some preferred a brief ten-minute check-in, while others described a session more comparable to a full therapy appointment.

Participants viewed these contacts as opportunities to review progress, revisit techniques, or receive light-touch support, but also as a space for relational connection and accountability:

*It would be good to have*,* maybe the odd follow-up call to say*,* if you done this*,* or for something for you*,* just to turn round and say*,* I used this technique this week*,* or it’s been a good week*,* or even have a laugh and a joke.* (PAT05)

We understood follow-ups as a way to serve dual functions: technical (refreshing skills, troubleshooting) and relational (being remembered, feeling held in mind). These suggestions reflect the value of structured continuity that reinforces maintenance without returning to full treatment.

Several participants recommended a ‘*buddy system’* (PAT16), pairing individuals with similar background upon discharge. Others proposed peer support groups or online forums involving ‘*people that have undergone CBT’* (PAT02). These ideas were driven by the desire for shared understanding and the reassurance of talking with others who “*had been through it*” (PAT16). We believe that participants’ emphasis on peer-based systems reflects a wish for normalisation, mutual recognition, and accessible support outside formal services, valuing learning from others lived experiences and fostering a sense of community and understanding. This aligns with personal-recovery models, where connection and shared experience can sustain motivation and technique use.

Two participants described the value of ‘*self-help apps’* (PAT01) for visualising and tracking progress. Others wanted easy access to information through a “*telephone helpline*” (PAT07) or a “*mental health website”* (PAT19) offering self-help materials, refreshers on techniques, and clear routes back into services. When suggesting support groups, helplines, or follow-up sessions, participants emphasised the need for a trained professional, ideally the therapist “*who followed you through the treatment*” (PAT23), due to established rapport and familiarity with their case. At the same time, participants acknowledged the importance of self-initiated help-seeking, noting that “*it’s up to the individual to… recognise the signs and respond in the appropriate way*” (PAT01). Participants pointed toward low-burden, on-demand tools that support independence while preventing skill decay. These suggestions reflect a desire for flexible self-management aids that reduce barriers to early help-seeking.

Some participants proposed mechanisms that simplify access and establish direct links with external service providers in the local vicinity re-entry into the service, including an independent referral route after discharge, or service-initiated links to external providers in the local area. *‘by the organiser contacting you*,* rather than me having to take that first step*,* that makes it easier*.’ (PAT21). These accounts demonstrate how administrative complexity can hinder re-accessing help. Participants valued processes that shift some of the activation burden from the individual to the system, especially during periods of vulnerability.

Data suggested that post-treatment care should involve various individuals in different capacities. This includes the GP, who is seen to have ‘*the first duty of care to the patient’* (PAT018), and thus should be aware of the treatment received and involved in any aftercare such as checking-in patients to see how they are coping. Alternatively, when patients suggested support sessions, online forums or helplines, patients viewed it essential to have a professional ‘*who was trained’* (PAT021) to moderate the sessions, and this would ideally be the therapist ‘*who followed you through the treatment*’ (PAT023) due to the rapport built and familiarity they have with the individuals’ circumstances.

Participants described a multi-layered vision of post-treatment care involving several actors. Some felt the GP should play a key role due to having “*the first duty of care to the patient*” (PAT18), including being informed about therapy received and checking in after discharge.

Participants envisioned collaborative maintenance: shared responsibility between the individual and service actors. Their emphasis on familiarity (continuity with the treating therapist) reflects the relational depth required for effective support, while the role assigned to the GP reflects a broader view of integrated care.

## Discussion

The present study explored patient perspectives on relapse prevention and the maintenance of treatment gains following low-intensity Talking Therapies (TT) for anxiety and depression. This qualitative analysis highlights the real-world complexity of sustaining recovery, the non-linear trajectory that many patients describe, and the mechanisms they perceive as enabling or impeding long-term well-being. We found that formal relapse-prevention initiatives are scarce and inconsistently implemented within and beyond TT services. Recovery maintenance is shaped by a dynamic interplay between contextual stressors (e.g., employment, health problems) and cognitive factors (e.g., memory). Conversely, sustained recovery is supported by robust support networks, appropriate medication use, and regular engagement in valued and pleasurable activities. Importantly, patient-generated recommendations centred on follow-up contact, clearer routes to external support, and stronger relational networks; all pragmatic service actions that can be interpreted within established change-maintenance theory.

### Enhancing integration of relapse prevention

Across services, relapse prevention is not standardised and usually it is primarily addressed with the use of booklets curated for relapse prevention distributed during treatment. Consistent post-treatment use of these resources appears to consolidate skills and reduce the likelihood of further intervention. Customised plans that synthesise therapy gains and anticipate setbacks are valued by patients [58–61], and findings support the use of online relapse prevention booklets within low-intensity treatment [61].

These findings align with relapse prevention and maintenance of change models, which frame relapse prevention not as a discrete post-treatment add-on but as an ongoing self-regulatory process involving self-monitoring, coping responses in high-risk situations, reinforcement of new habits, and timely adjustment after lapses [62]. Participants’ emphasis on continued access to materials and structured contacts aligns with maintenance of change models, highlighting these mechanisms as influences on self-regulatory cycles and as factors that strengthen self-efficacy. Similarly, the Transtheoretical Model of behaviour change [33], conceptualises relapse as part of a cyclical process and supports stage-appropriate maintenance strategies. Although these models originated largely in addictive behaviours, their principles translate well to common mental health conditions in primary care and self-management contexts [41, 63].

This makes them highly relevant frameworks for interpreting the present findings in TT services. Our findings illustrate how post-treatment barriers, such as difficulties sustaining use of therapeutic techniques, could be addressed through interventions aligned with these models. The structured consolidation phase, a recognised element in models like the BCW and central to relapse prevention in substance use disorders, might prove beneficial to anxiety and depression treatment. By systematically incorporating this defined phase for reinforcing skills and planning for relapse, low-intensity anxiety and depression services could significantly enhance the long-term maintenance of treatment gains. This represents a clear opportunity for future service development. Findings revealed a lack of emphasis on targeting relapse post-treatment. Data highlighted therapists placed great importance on revisiting treatment paperwork and re-referring to the service as the only relapse prevention initiatives. Although a core component of preventing relapse includes continued use of the skills learnt during treatment [40], providing additional support beyond treatment would further assist in maintaining recovery. Recommendations within the TT manual [45] and low-intensity CBT guides [61] emphasised incorporating follow-ups to identify and address early signs of relapse. However, the present study did not provide any evidence on this aspect. Research exploring relapse prevention interventions demonstrated their benefits in preventing relapse [40, 64, 65].

One of the core findings emerging strongly from our findings was the importance of supporting patients to external providers to address physical health problems, financial stressors, or unemployment issues, thereby extending the reach of relapse prevention beyond symptom management and supporting whole-person care. Better integrated pathways between TT and external providers are associated with more coordinated care and more efficient service use [66–68]. Our findings suggest that patients would benefit from clearer, more proactive connections/plans to these supports during and after treatment. Improving links between TT services and primary care aligns with policy priorities such as the NHS Five Year Forward View for Mental Health [69].

Figure 1 illustrates the collaborative nature of recovery across patients, TT services, and external providers. Strengthening these connections, particularly around post-discharge transition, may increase the likelihood that skills are maintained and barriers are addressed in a timely manner.

### The obstacles following treatment

Participants reported substantial barriers to maintaining wellbeing, especially work-related stressors and co-occurring health conditions. Employment-related adversity is associated with elevated anxiety and depression, and unemployment is a risk factor for anxiety disorders [38, 70–72]. Embedding employment advisors within TT services can improve outcomes [63, 72], but patients also require broader linkage to physical health, social, and community resources to achieve holistic care [73].

Difficulties remembering and continuing to implement treatment techniques were common. This is consistent with evidence that patients often recall treatment content poorly [74–76], which may increase relapse risk [77]. Materials provided during treatment can act as external memory supports [75], and brief prompts (e.g., periodic text messages or app-based nudges) can cue skill use in daily life and help sustain behaviour [78]. Framed within self-regulation theory, these aids function as feedback and cueing mechanisms within a maintenance cycle rather than as ancillary reminders.

### Value of support networks

Findings reinforce the proactive role of social support networks in sustaining recovery. Support from family and friends is associated with lower depression and anxiety [40, 72, 80–82] and facilitates social aspects of recovery [30]. Future relapse-prevention efforts in low-intensity treatment should consider access to and integration of support networks -including peer groups or “experts-by-experience”- to create a sense of belonging and shared learning [83]. Online forums or buddy systems may be particularly valuable for individuals with limited natural supports, and narrative accounts can normalise setbacks and model adaptive coping [85].

### Recovery as a journey

Figure 1 depicts the non-linear trajectory many participants described. Treatment equips patients with techniques, but sustained use varies across contexts and over time. Qualitative research similarly shows that patients understand recovery as long-term and expect occasional setbacks [40].

Research on personal recovery further underscores the individuality of the recovery journey, echoing patients’ views of recovery as a process of regaining control over problems encountered throughout their lives [84] rather than being exclusively associated with symptomatology.

The Personal Recovery Framework (CHIME: Connectedness, Hope, Identity, Meaning, and Empowerment) helps explain why patients emphasised social connection, collaboration, and regaining control as important processes underpinning durable change [29]. Although we defined relapse clinically (consistent with NHS TTad services), functional and subjective perspectives are also salient. Future research should explore how clinical, functional, and personal recovery align or diverge when monitoring long-term outcomes.

### Recommendations to support progress after treatment

Our sample included individuals meeting recovery criteria at the end of low-intensity treatment within TT services and with varied post-discharge durations, which strengthened the diversity of perspectives. Furthermore, using the CFIR framework facilitated systematic examination of multi-level factors shaping post-treatment maintenance and highlighted concrete implementation targets for services.

Recommendations from the findings can be grouped into two broad categories of actions that could support sustained progress following low-intensity TT. The first set reflects core clinical maintenance practices that are well-established within relapse-prevention and self-regulation models, yet were delivered inconsistently in routine care. The second set represents more novel recovery-oriented strategies, emphasising social connection, community belonging, and broader wellbeing domains often overlooked in clinical definitions of relapse but central to personal recovery frameworks as highlighted by patients within our study.

#### Core clinical maintenance practices (expected but inconsistently delivered)

Participants highlighted substantial inconsistencies in relapse-prevention provision, with some receiving minimal discussion or a booklet, while others were discharged without any prior indication or structured preparation. Three recommendations relate to essential maintenance practices that, although standard in theory, were not reliably implemented: (1) embedding an end-of-treatment maintenance plan that identifies personal relapse signatures and coping strategies; (2) offering a brief follow-up or booster contact to reinforce skills and troubleshoot emerging difficulties; and (3) providing optional, low-burden prompts (e.g., app) to cue continued use of therapeutic strategies. These actions align with self-regulation models, where feedback, reinforcement, and timely reminders facilitate skill retention and adaptive coping over time.

#### Recovery-oriented innovations (novel contributors to long-term wellbeing)

Beyond clinical maintenance, participants emphasised needs consistent with broader definitions of recovery, including social connection, identity reconstruction, and support during transitions. Three recommendations reflect these more innovative and recovery-oriented priorities: (1) strengthening social support networks through peer forums, buddy systems, and opportunities to connect with experts-by-experience; (2) enhancing the transition from services to community resources through more visible, accessible, and better-coordinated information pathways; and (3) promoting wellbeing beyond symptom reduction by facilitating engagement in meaningful activities and supporting identity, purpose, and connectedness in line with the CHIME framework. These recommendations extend relapse prevention beyond clinical symptom management and recognise that maintaining wellbeing involves navigating daily life, relationships, and community environments.

The implementation gaps observed — including variable discharge preparation and limited proactive maintenance support — are common across international mental health systems, indicating that our findings have utility beyond the UK context. The proposed recovery-oriented recommendations align with international frameworks promoting community integration, social connectedness, and wellbeing beyond clinical symptom reduction, suggesting clear applicability to global mental health systems.

## Limitations

Nonetheless, there are limitations which require discussion. Firstly, a key limitation of this study is the lack of diversity within the sample, which was predominantly White. Evidence suggests that individuals from White ethnic groups are more likely to receive treatment following assessment compared to other ethnic groups [85], and that ethnic minority groups often experience poorer treatment outcomes [79, 86]. This underrepresentation may therefore skew understandings of recovery experiences and limit the applicability of findings to more diverse populations. These disparities highlight the importance of culturally competent care, whereby healthcare providers actively address cultural differences, biases, and systemic barriers that shape engagement with services and influence recovery. Future research should prioritise inclusion through targeted recruitment strategies to ensure representation across ethnicity, religion, sexual orientation, and other underrepresented groups, to examine whether their recovery experiences align with or differ from those of the current sample.

Secondly, our sample comprised mainly those who had no experience of relapse following discharge at the point of data collection. The few who demonstrated a relapse in the present sample shared many similarities with those who did not. However, considering the small sample, it is difficult to ascertain whether other individuals who relapse following treatment have shared experiences. Therefore, research targeting individuals who have relapsed would provide additional insight into their experiences.

Thirdly, although the mixed and pragmatic recruitment approach enabled the inclusion of participants from multiple services, it may also constitute a limitation of the present study. Employing different recruitment strategies across sites may have introduced sample variability, potentially affecting the representativeness of participants and the nature of their accounts, which, in turn, could affect the transferability of the findings. To mitigate this limitation, future research should implement a standardised recruitment protocol across all sites to minimise sampling variability and enhance the methodological rigour and transferability of results.

Finally, another limitation of this study is the lack of direct assessment of treatment fidelity. Since sessions were not recorded, we cannot determine how variations in practitioner adherence to the care model (i.e. Guided Self-Help based on CBT principles following NICE guidelines) contributed to inconsistent patient experiences. Furthermore, data from a related qualitative study exploring practitioner perspectives on relapse prevention [87] highlighted that service pressures likely constrained the time available for thorough relapse prevention planning. This previous work also highlighted the absence of standardised approaches to relapse prevention, limitations on available resources, and the need for further training and an approach to recovery beyond clinical symptoms. Collectively, these documented service-level constraints and gaps also help to explain the observed variability of care and differences on patient experiences identified in the present study.

## Conclusion

The present study explored patient perspectives on relapse prevention and the maintenance of treatment gains following low-intensity Talking Therapies (TT) for anxiety and depression. This qualitative analysis revealed two interconnected but distinct domains shaping long-term recovery. First, patients described gaps in core clinical relapse-prevention practices—such as structured discharge preparation, opportunities for consolidation, and consistent post-treatment follow-up—that were inconsistently delivered and contributed to variable experiences at the point of discharge. Second, participants highlighted a broader set of recovery-oriented needs related to sustaining wellbeing beyond symptom reduction, including access to meaningful social support, smoother transitions from services to community resources, and support for living well in everyday contexts. These findings emphasise that maintaining recovery requires both reliable clinical maintenance processes and attention to the personal, social, and contextual factors that shape life after treatment, pointing to two complementary areas for service development.

Future phases of the CO-IMPROVE project will refine these insights into practical tools and evaluate their feasibility in clinical settings. Although conducted within NHS Talking Therapies, the mechanisms identified—including self-regulatory maintenance processes, memory-related barriers, and the importance of social networks—are internationally relevant and align with global trends in scalable psychological interventions. As many countries expand access to brief mental-health treatments, the recommendations offered here provide transferable insights to strengthen long-term outcomes and support recovery beyond symptom reduction.

## Supplementary Information

Below is the link to the electronic supplementary material.

Supplementary Material 1

Supplementary Material 2

Supplementary Material 3

Supplementary Material 4

## Data Availability

The data set generated and analysed during the current study is not publicly available due to privacy and ethical restrictions (i.e. potential for breach of anonymity) but is available from the corresponding author upon reasonable request.

## Abbreviations

CFIR: Consolidated Framework for Implementation Research
CO-IMPROVE: Co-developing Improving Access to Talking Therapies for anxiety and Depression
GAD-7: Generalised Anxiety Disorder-7
NHS: National Health Service
PHQ-9: Patient Health Questionnaire-9
PPI: Patient and Public Involvement
PWP: Psychological Wellbeing Practitioners
ROM: Routine Outcome Monitoring
TT: Talking Therapies
WSAS: Work and Social Adjustment Scale
BCW: Behaviour Change Wheel
