# Supplementary material for: How can patients with anxiety and/or depression maintain wellbeing following discharge from low-intensity interventions? A qualitative exploration of patient perspectives
Source: BMC Psychiatry. 2026 Jul 11;26:762. doi: 10.1186/s12888-026-08135-5 (PMC13647811; doi:10.1186/s12888-026-08135-5)
Supplement: Supplementary file 1 [file 12888_2026_8135_MOESM1_ESM.docx]

**Supplementary File 1: Appendix A**

**Semi-structured Interview Schedule**

**INTERVIEW SCHEDULE – PATIENTS**

**Interview objectives:**

1) To explore barriers and facilitators influencing recovery (i.e., maintenance of treatment gains) and relapse (i.e., periods of deterioration) following low intensity interventions for depression/anxiety in IAPT
2) To explore difficult periods of time following treatment and how those were (or not) overcome (e.g., what helped/went wrong, resources/support)
3) To identify what is the care/support/resources needed following treatment to maintain recovery and/or address relapse in a timely manner

*This is a list of topics, questions and prompts to guide the conversation with patients during the interview. All questions may not be asked or discussed, and the questions may be adapted and phrased differently depending on the participants and the natural flow of the conversation.*

This interview schedule will be reviewed and piloted with our patient and public engagement advisory group and would be amended following feedback/recommendations.

*Please note this guide could be slightly modified over time following preliminary analysis during data collection.*

**Preamble**

- Reminder of the purpose of study
- Explanation of ethics, consent, and confidentiality
- Structure of the interview (may be some overlap in questions and responses, remind participant it is up to them to answer the questions and it is OK if they do not want to reply)
- Allow opportunity for questions prior and during the interview
- Remind participants about the recording
- Participants should have completed the Consent Form prior to the meeting, if consent has not been taken, record consent verbally in a separate recording

[Start recording]

A. **EXPLORING THE IAPT TREATMENT RECEIVED**

Thinking about the psychological treatment you have received in IAPT:

1) Would you be able to briefly describe the treatment you received? (e.g., symptoms, focus of treatment). Explore pending on response.
2) What would you say you have gained from treatment? What were the benefits from the treatment you received? Could you please describe and expand.
3) What were the key things you have done during treatment to achieve recovery/overcome your [select pending on treatment: low mood/anxiety]?
4) In terms of end of treatment, what are you able to recall from the last session/s? Please describe and provide an example to illustrate that.
5) What were the conversations (if any) with your therapist about your achievements during treatment and how that translated into “achieving recovery”?
6) How did you feel about ending your treatment? What were your thoughts/concerns (if any) at the point of discharge? How have those been addressed?
7) What were the recommendations/discussions (if any) you had with your therapist to maintain recovery and prevent relapse?
8) What conversations can you recall about what to do if you were feeling low in mood /highly anxious following treatment?
9) What did you know about different types of supports you could receive following treatment?

- Where/who did you receive information from?
- How did you receive the information? (e.g., what format: verbal, written, visual)
- How easy/difficult was it to find/get the information? (e.g., If it was not easy, how could it be made more accessible?)
- When did you receive the information?
- What did you think about the information? (e.g., make sense/was it clear?
- Would you have liked any additional information?

**B**. **EXPLORING EXPERIENCES FOLLOWING TREATMENT**

Now, I would like to ask you about your experiences following treatment. So, since you have completed the treatment in IAPT:

10) How have you been feeling? Would you say you have been able to maintain the benefits of your treatment?

- If Yes, please describe and provide examples.
- If No, please describe and provide examples. What do you think it has interfered in maintaining your recovery? What could have influenced/triggered that? What would have been helpful at that point from a supporting perspective?

11) Pending on the response to the above question, you could take the opportunity to use the clinical information gathered from the questionnaires participants have completed prior to the interview and indicate if this is reflected or not in the scores of the questionnaires. If questionnaire scores differ from patient’s perspective, please explore why this could be?

- I am aware you completed a few questionnaires prior to the interview and provided valuable information about yourself. Most of the questionnaires you completed were the same you completed during your treatment. I had a look at those questionnaires and they indicate:

-you have been/not have been able to maintain recovery for [select as appropriate: symptoms of depression and/or anxiety]. This is in line/not in line with what you mentioned, what do you think about that? [Disagreement could be related to the point of completion of questionnaires, meaning that maybe the patient maintained recovery for 3 months and the last month or so did not]

12) What were the challenges you have faced to maintain recovery following treatment?
13) I anticipate there might have been difficult times since you completed your treatment and I wonder how did you manage (or not) those times/situations? What have you done? What helped/what did not help?
14) How were you supported following the completion of your treatment? Please describe and provide examples. [Link this to Question 9 if it makes sense]
15) Based on your experience, what would you change (if anything) from the end of your treatment? (e.g., what would you say would be important to do/know in your last session/s)

**C**. **EXPLORING VIEWS TO UNDERSTAND/IMPROVE CARE FOLLOWING TREATMENT**

16) How do you feel/what do you think about current programs/practices related to post-treatment care?
17) Based on your experience, what could be done differently to help people following treatment to:

- maintain treatment gains for a long periods of time (e.g., over 6 months)?
- prevent relapse?
- overcome relapse in a timely manner?

18) What type of care/support is needed to maintain recovery following treatment and potentiate those gains (i.e., ensuring changes made during therapy transition to lifelong skills)?
19) What could be done to easily identify relapse following treatment? And/or address relapse in a timely manner?
20) If there was a possibility for offering care following treatment for those that have achieved recovery at the end of treatment to maintain treatment gains/prevent relapse, what would you say would be important to take into consideration towards that end?
21) What are your expectations for the “post-treatment care”?
22) What would be the key elements that you would think should be available to patients and in which form (e.g., written, online, face-to-face, group) to maintain recovery an potentiate treatment gains over time? Who should deliver those? Frequency? (what, who, how, frequency)
23) Taking on board responses to question 21, ask about how the proposed suggestions for after-treatment care compares to the after-care received? What are the anticipated benefits if the proposed suggestions where to be in place? How could patients already in recovery could be made aware of this?
24) Taking into consideration responses of this section of the interview (Section C), how essential is it to have an after-care programme in place? Would that meet the needs of most patients?
25) Is there anything else that you would like to add?

To close the interview:

Thank you very much for participating in the interview, all the information you provided is of much value and I would like to thank you once again for your time today. I am stopping the recording now.

[Stop recording]
