# Supplementary material for: How can patients with anxiety and/or depression maintain wellbeing following discharge from low-intensity interventions? A qualitative exploration of patient perspectives
Source: BMC Psychiatry. 2026 Jul 11;26:762. doi: 10.1186/s12888-026-08135-5 (PMC13647811; doi:10.1186/s12888-026-08135-5)
Supplement: Supplementary file 2 [file 12888_2026_8135_MOESM2_ESM.docx]

**Supplementary File 2: Appendix B**

**Co-developing Improving Access to Psychological Therapies (IAPT) services to improve long-term benefits for patient with depression and anxiety**

**Post-treatment Experiences Survey**

The completion of this questionnaire is optional, meaning that you do not have to complete it if you do not want you.

The aim of this survey is to allow you to provide information about your experiences following your treatment. It could take between 5 to 10 minutes to complete.

1) Thinking about the treatment you have received, what would you said you have gained from completing this treatment to achieve recovery?

________________________________________________________________________________________________________________________________________________________________________________________________________________________________________________________________________________________________________________________________

2) How did completing the treatment benefit you?

________________________________________________________________________________________________________________________________________________________________________________________________________________________________________________________________________________________________________________________________

3) What were the plans/strategies (if any) agreed with your practitioner to maintain your recovery and prevent any potential episodes of relapse and/or to deal with difficult periods?

______________________________________________________________________________________________________________________________________________________________________________________________________________________________________________________________________________________________________________________________

4) What were the challenges you faced after completing your treatment?

__________________________________________________________________________________________________________________________________________________________________________________________________________________________________________________________________________________________________________________________

5) How were you supported following the completion of your treatment?

____________________________________________________________________________________________________________________________________________________________________________________________________________________________________________________________________________________________________________________________

6) What would be your recommendations/suggestions of the care you or others may need/benefit from following treatment?

________________________________________________________________________________________________________________________________________________________________________________________________________________________________________________________________________________________________________________________________________________________________________________________________
