# Supplementary material for: How can patients with anxiety and/or depression maintain wellbeing following discharge from low-intensity interventions? A qualitative exploration of patient perspectives
Source: BMC Psychiatry. 2026 Jul 11;26:762. doi: 10.1186/s12888-026-08135-5 (PMC13647811; doi:10.1186/s12888-026-08135-5)
Supplement: Supplementary file 3 [file 12888_2026_8135_MOESM3_ESM.docx]

**Supplementary File 3: Table 2**

*Summary of CFIR constructs, definitions and illustrative quotes.*

| **Domain** | **Constructs/Subconstructs** | **Description** | **Quotes** |
| --- | --- | --- | --- |
| **1.Intervention Characteristics** | Relative Advantage | Patients described advantages and benefits categorised into two subconstructs below, one referring to advantages experienced through treatment, and the other related to anticipated benefits/advantages from receiving support following treatment. | N/A |
|  | - *Treatment** | Patients stressed the benefits they received from undergoing treatment including the ability to complete mundane tasks, returning to a pre-illness state and becoming more sociable. | So part of the CBT that was most useful was seeing things in proportion. I live on my own and when you’re not feeling very well, it’s difficult, it’s very much all or nothing. You tend to catastrophise things and the CBT taught me not to do that, to go, okay, well, today has been a bit rubbish, but that doesn’t mean tomorrow is going to be rubbish. PAT02 |
|  | - *Post-treatment care** | Patients reflected on the potential benefits of receiving support following treatment, emphasising how providing additional support can help sustain recovery and identify instances in which individuals are heading towards another episode earlier, particularly if they struggle with introspection. | But I suppose, some people might not be able to see that they are doing...the way that they might not be able to pick up on how they are feeling, so they might not then realise to then rerefer. So, I think having, like, check-ins or, maybe, for, like sort of, more vulnerable people then that might be beneficial. PAT10  So, if there are some links that could be shared, so for people to self-refer, or types of treatments offered, that would be good, because if someone needs it, they could just go back to the email and find it easily. Perhaps that would be good. PAT19 |
|  | Adaptability | Few patients provided examples of ways in which the treatment had been tailored to address their unique needs and experiences e.g., unlocking relevant modules for online CBT treatment. | It’s not a one size fits all, it’s much more tailored, this particular CBT that I’ve done. And that’s why I thought it was so helpful. PAT02  I really liked how I was able to have an appointment at half eight, which massively helped. Work did say that I would be able to take time off of work, you know, to do them. But again, someone, if they didn’t want their work to know or, you know, struggled certain times with a typical nine to five, the fact that I could do half eight ‘til nine was perfect for me. PAT34 |
|  | Complexity | Patients highlight and acknowledge the difficulties associated with completing questionnaires during treatment, including the time they are completed and the limited response options. | I find it a bit difficult sometimes to quantify how you feel, like, in this, yeah, it’s not always easy to quantify in a questionnaire. Of course, yeah, it’s a way to measure some things but it doesn’t really translate always, accurately. PAT23  I would say there were definitely days where I felt bad, but based on my scores, the therapist said he thought I was feeling good. So I would say they don’t always accurately reflect, I would say. PAT29 |
|  | Design Quality and Packaging | Patients share their perceptions of the diverse treatment modalities (Online, face-to-face and telephone), and the different formats in which they received information (physical copies, electronic copies) to assist with their recovery during and following treatment. | And that [Questionnaires] did work in part, but it’s very easy when you’re not seeing someone to cheat, and, you know, if you’re not seeing someone face to face, it’s very easy to say on the phone, yeah, I’ve done all that, when you haven’t really. PAT21  The thing is as I’ve got older concentrating on written material is probably more difficult for me than it used to be then. I don’t know. PAT27 |
|  | *Intervention context** | Patients provide detailed description of the treatment they received (including duration, referral route, modality etc.) and the discussion they had with their therapist during treatment to reach recovery.  Further information about the content of the treatment sessions included descriptions of skills and techniques learnt which is explored in the following subconstruct. | I had six half hour telephone appointments with a therapist. Each week I would fill in all the forms about how I was feeling, usually online before the appointment started. We’d also set goals and actions to take for the next week, and that was, it was six appointments every two weeks thereabouts, it finished in September. PAT21  So, I went to my GP and then they gave me a link to talking therapies. So, I got in touch with talking therapies and then that instigated a series of video meetings, like this, Zoom, on a fortnightly basis or maybe a bit longer than that, depending on the circumstances. Those sessions lasted typically about 40 minutes or something like that. PAT28 |
|  | - *Techniques** | Patients described the assortment of techniques they learnt during treatment to aid their recovery. | But there was the distraction. And then you'd have worry time. So you would give yourself 5 min or 10 min to worry about something, and then put it to bed. Then there's rumination. Don't ruminate. And also, one that was for me at the particular time was I was setting myself unrealistic targets.PAT05  I know she used some analogy with a courtroom and putting negative thoughts on trial really and making…I thought that was good. And problem solving through stuff, which, yeah, I thought that was helpful. And identify triggers as well. PAT20 |
| **2.Outer Setting** | Cosmopolitanism | Few patients mentioned how the service was linked with external services from sending the discharge letter to the GP to connecting patients with services to aid other aspects of the health (e.g., physical) or finances (advisors), topics not covered within treatment. | All I’ve had since treatment finished, was a phone call off my doctor's asking how I was doing. Basically that's it. That's all I’ve.. I am also seeing a job advisor through the National Health through things, so I still see him from time to time. Well, obviously that's nothing to do with the mental health side. Well, it is in a way something to do with the mental health side, but it's not something we discuss, it's about finding employment now. PAT04  I think it would be good if it was a little bit more linked to GPs. PAT02  I think there was a letter sent to my GP from the therapist just to say this has been signed off. But I think it was like if I went with any more issues, then I would know that my doctor would see that I’ve had that treatment and stuff. PAT14 |
| **3.Inner setting** | Structural characteristics | Few patients commented on the speed of the service when trying to access support. | So, I just think well…but then it's the access to it, because it's lots of waiting, isn’t it? I was very lucky actually I got mine fairly rapidly somehow, I don’t know how that happened, but I was spoken to within a couple of weeks. Whereas I know some of my patients are waiting months. And I think that makes a massive difference, because obviously it's something that needs to be implemented when you're unwell. PAT20 |
|  | Culture | Some patients reflect on the organisation's values, specifically the significance placed upon shared decision-making and collaboration through adhering to patient choices about their treatment. | And she gave me the option really, she said, now we could continue you this if you like, or if you wish we can just stop it there. It was four sessions and I felt happy enough stopping it. PAT11  It was mutually agreed because I felt the same, that attending another session was probably just taking up space that somebody else needed more than I did at that time. PAT32 |
|  | Implementation Climate: | This construct is divided into the following subconstructs as per the CFIR. | N/A |
|  | - Tension for Change | Patients highlight the problems with the lack of post-treatment support demonstrating a strong need for such innovations to be included within mental health care. | It’s like with anything, you can’t just…people to go through what they’ve been through and then just, that’s it, cold turkey, off you go. Because there’ll be so many people that relapse, because there isn’t easily available care for the person to speak to, or whatever, so yeah, I think for everybody, it would be beneficial. PAT16  Yeah, the problem is that with that particular service it’s, you cannot really get, let’s say, immediate help. So you have to refer yourself and then you go on a waiting list and then they contact you and then you book an appointment. So that took, like, a couple of months to be honest with you, when I did it this time. PAT23 |
|  | - Relative priority | Patients perceive post-treatment care with differing levels of importance based on personal experiences following discharge, pending on their experiences following discharged | I think it’s very important because personally, I don’t consider myself cured from anxiety and I don’t think I will be, so there are always ups and downs. So, if there is some way that the person can maintain these tools rather than just doing it for a period, I think that will be very beneficial to prevent any… I mean, it’s not possible to prevent it, it might happen anyway, but it’s easier to prevent recurrence with a more regular tool that they can use. PAT19  Again, I think that there is other tools that people could use if they didn't receive this aftercare and they just went back to self-referral or back to the GP. So, it's probably not super important. PAT14  I think it’s quite essential in general to focus on, I mean, follow up treatment for me would be something like preventative care, as well. You do a session, you do a course of sessions, then you are discharged, but then if you’re not followed up on you might reach a point where you escalate. And then it’s not preventative anymore, you have to go into a, kind of, a crisis management. PA23 |
|  | - Goals and Feedback | Some patients highlighted how the treatment aligned with and addressed their purpose of seeking treatment initially demonstrating the suitability of the treatment. | I know it's there if I need it, but I think more people need to know about this, even though the doctors put you on to it, if you're getting messed kind of state. PA04  I went there for like guidance and I felt like I got good guidance because they gave me tools that I didn’t have, so I thought in that time in my life, it was a good place to have gone, it was someone to sit there and someone to listen to me. PAT25 |
|  | Readiness for implementation: | This construct is divided below as per the CFIR with additional sub-constructs outside of the CFIR added. | N/A |
|  | Available resources | Patients described the resources received during treatment which they have used following treatment. | But I think being able to access the SilverCloud is really good, it's quite nice knowing that that's just there if I need it as another tool. PAT14 |
|  | - *During Treatment** | Patients described the different resources received during and throughout treatment sessions including links to other resources. | I think we also discussed, sort of like, techniques we’d used throughout the sessions but also, she gave me, sort of like, other...what’s the word, like, not literature but other, sort of, links and, sort of, things that I could use, sort of, day-to-day. That I could log into. PAT10 |
|  | - *After Treatment** | Patients emphasised the self-referral option as an available resource as the main support following treatment. One patient explored the accessibility of online CBT resources available for a year from discharge. | I think what she said was, basically, it’s there and I can access this for a year after the start of the treatment. So I’ve been through and I’ve had a look, I’ve downloaded more of the meditations and those are on my phone now. So it’s nice to be able to go back and do that. PA02  Again, it was really if I needed to go back to go back to somebody or refer to the paperwork. PAT05 |
|  | Access to knowledge and information | This subconstruct has been further categorised below to differentiate between knowledge obtained during and after treatment. | N/A |
|  | - *During Treatment** | Patients commented on the ease of and difficulties in accessing information about the treatment and the access to digestible information provided by therapist during treatment. | I think she gave me all the information that I actually, you know, it was all presented to me, any help I thought I would need for future. PAT25  I’ve actually still got the email, the notes and things so I can refer back if I need to. PAT07 |
|  | - *After Treatment** | Ease of access to digestible information about support available following treatment received after discharge. | They do point you in directions of different services that are available, but you’ve gone first of all to that service and that’s the one that you look at as helping you recover. I suppose it’s a bit like being an alcoholic and you go four times, okay, you don’t drink anymore, where are you three, six, 12 months down the line to stop you from relapsing, and there’s nothing in place really, unfortunately, at the moment. PAT25  I maybe would have been better off going to the library and getting a book out, which is probably something I would do, the library does have a very good section of mental health information books that they have in a separate section – my local library is very good. PAT27  But yeah, they were really good at advising me who to contact, gave me a number of a good place to contact [counselling service] that’s local. PAT32 |
| **4.Characteristics of Individual** | Knowledge and belief about intervention | This construct explores the attitudes and values placed upon the intervention and participant’ familiarity with facts about the intervention. This is split below. |  |
|  | - Knowledge Relapse prevention* | Patients describe a lack of knowledge and familiarity with post-treatment care options currently available. | But I mean, a relapse could happen…if you like, I had a relapse after 23 years and you couldn't expect a GP to be looking at you all the time for that time, so there has to be a time limit on it. PAT11  I don’t think there really is any post-treatment, you just have to start the process again. PAT16 |
|  | - Beliefs Relapse prevention* | Patients’ perceptions about relapse and relapse prevention following discharge, including the process of recovery. | It’s a bit more complicated than that. There isn’t an immediate cure. I think it’s one of the things, especially with mental health, that you have to keep at. It’s not just, oh, I had the CBT, I’m totally cured now, this is great. It’s about keeping on track. PAT02  I think the biggest thing is, we all have bad days, so if you have a bad day, it’s not a bad week, and it’s not a bad life, there’s always tomorrow. So, you can always start again. I think that is the biggest thing. PAT21 |
|  | Self-efficacy | Participants’ confidence in their capabilities to successfully continue with their recovery journey independently with the tools provided during treatment. | All these trials and tribulations, I’m finding ways to get over them. PAT03  But I've…I have taken away a lot from the last session as well. As in right, although I'm not getting face to face, this doesn't mean I've got to stop doing what I've been doing. PAT04  Possibly but I think I was just feeling well and ready just to crack on with life really. I don't think the counsellor could have given much more. PAT20 |
|  | Individual stage of change | The stage of change in which patients are in before, during and after treatment according to the stages of change model. The treatment is the action stage and remaining in recovery following discharge will be the maintenance phase, and if patient experienced a relapse following treatment it will be the relapse phase. | And that's just the way I am now. People can't believe how I’ve changed. Well, they can believe it because I'm talking to them, but they’ve seen a vast improvement. PAT03 (*Maintenance stage*)  But I know this time with me I realised in early July that I was going downhill and I went to…I sought treatment immediately, whereas the first time it happened, in [year] , I didn’t know what was going on so I just thought I was maybe going mad. PAT11 (*Action stage*)  Oh, very up and down. Yeah. First of all I thought I was going to be absolutely fine, but my…I realised my anxiety is just very day to day, and, you know, some little tiny thing goes wrong and I feel completely flummoxed. But I’ve not been feeling anywhere near as bad as I was at the latter end of last year, nowhere near, no. But I am up and down a lot. PAT27 (*Maintenance stage*)  Okay most days. I’d say there are days where it’s still not great but I don’t spiral as much as I was prior, so I’d say better but not 100 per cent. PAT29 (*Maintenance stage)* |
|  | Other personal attributes | Few patients described personal traits such as motivation and capacity, explaining an enthusiasm to share their learning with others. | So my mind is going more of…my journey feels more that I don’t kind of need a lot of support but I think it’s my time to give and help. I’ve done it for 30 years in a job and I want to do it for a wider society, yeah, that’s where I’m at. PAT25  I think early on, it was still the feeling of, can I be bothered with this, early on it was, is this making any difference, is it going to make any difference, and does it matter if I fill these things in? But then, as the time went on, it’s thinking, well yes, it does matter, and it is working. So that was the motivation to do it. But initially it was kind of, do you know, is there any point? But that was how I was feeling about everything anyway. PAT21 |
|  | *Meaning of recovery** | Patients’ perceptions of the recovery journey and what being recovered means for them. | Day by day, I think. It’s taking a day at a time. PAT07  Well, I’d define recovery as I’m just feeling an awful lot better, you know, I’m more interested in doing things, I just…really at the time, I didn’t want to do anything.PAT011  I think just enjoying my life a bit more, and just feeling more like myself. PAT027 |
|  |  |  |  |
| **5.Process** | Planning | Patients’ suggestions for post-treatment care initiatives to address relapse following discharge and what they would entail. | So I don’t know, maybe a group like that, of people that have undergone CBT that might want to talk about their experience. PAT002  I think the personal contact would be in an ideal world a good thing just like a telephone helpline.PAT007 |
|  | Engaging | Patients described the various types of individuals involved in the delivery of the treatment interventions and any potential individual to be considered in suggestions for post-treatment care. This has been divided below. | It could come from community. It could come from family and friends. It depends. You know, some people are very reluctant to share feelings, I’d probably put myself with them at the start because we realised real benefit of talking things over with people. So, you can talk things over with professionals. But talking over with friends, work colleagues, lots of different people can provide support. PAT001 |
|  | Formally appointed internal implementation leaders | Individuals within the organisation who have been appointed with responsibility for implementing the intervention. | But also, you know, even if it was, sort of, I don’t know, like a duty worker, someone that just follows up and has a bit of a brief of a read of my file and just saying that I wanted to check…I wanted to ring you for a check-in I see that you had it, how’s things been going? You know, just to have that touch base if it was felt that it needed a little bit more of an understanding of the work. PAT034 |
|  | - External Change agents | Individuals affiliated with an outside entity who formally influence, facilitate or are involved in intervention decisions. | But I would like to…I’m sure it would be good to keep up the sessions with the GP or somebody…with somebody for a year at least, maybe a bit longer and then maybe ease it off if you felt that…you felt sure that there wasn’t going to be a relapse PAT011  In an ideal world, and probably if you go back 20 years, because I can’t even remember which GP as well, I don’t know; they’re all really nice but they’re different people every time I speak to somebody, so I’m not actually sure which one it was that I spoke to about this problem. In an ideal world they would ring me back, in a…and then say, how are you getting on, are you still on, you know, are you still on [inaudible]? PAT027 |
|  | - *Patients** | Description of how individuals who use the service can be encouraged to engage with the innovation and continue implementing their learning beyond the setting on their own. | I just think it's quite difficult to ask for help and I think if you’ve been signed off from your therapy, it might be quite hard to say actually, I don't think I should have been signed off or actually I'm still struggling. Whereas if there's a session in to double check that and say like you were signed off, are you still feeling good? Are you still managing then? Then I think it would open up a bit more of a conversation. PAT014  Again it depends on the individual because some people might be more intuitive about their own, kind of, state. And some might be a bit more, you know, resistant to acknowledge that, yes, I’m not doing well and I need help. PAT023 |
|  | - *Professionals** | Individuals who are responsible for delivering the intervention to service-users i.e., the therapist. | But that being done maybe by the person who did it originally. Because although we’ve exchanged emails, we’ve not met before today, so in the nicest way, you don’t know what bad looks like, whereas she definitely would.PAT018  I think it should not just come from the service. I think it should come from the person that's been counselling you ideally, that therapist because it just gives it that continuity, you know that they know about your situation. PAT020  Because sometimes some people work better with people that they know. PAT034 |
|  | Execution | Carrying out or accomplishing the implementation according to plan. | But financially, and looking at that, online would be the cheapest option, I would think, for people to be in touch with people. PAT021  But again we’re talking about money and facilities and people being able to do that.PAT026 |
|  | Reflecting and evaluating | Qualitative feedback about the progress and quality of intervention. | There’s nothing I would change, I thought it was all very positive, brilliant. PAT003  Well, at first I thought it was a waste of time and then at the end I thought it was very good, really. PAT011  So I think as I look back in hindsight, the CBT was absolutely the right method. The timing worked very well. It was definitely helped by the fact, medically, I was starting to see some improvements. So maybe I was more willing to accept, and in some ways, I hadn’t got as much going in. PAT018 |

*Note*. ***** Represents newly developed constructs within the CFIR. N/A =Not available
