# Supplementary material for: How can patients with anxiety and/or depression maintain wellbeing following discharge from low-intensity interventions? A qualitative exploration of patient perspectives
Source: BMC Psychiatry. 2026 Jul 11;26:762. doi: 10.1186/s12888-026-08135-5 (PMC13647811; doi:10.1186/s12888-026-08135-5)
Supplement: Supplementary file 4 [file 12888_2026_8135_MOESM4_ESM.docx]

**Supplementary File 4: Appendix C**

Illustrative worked example of DEDUCTIVE data analysis (Raw Data 🡪 Deductive CFIR construct 🡪 Analytic summary 🡪 Theme)

| **Raw Data Excerpt** | **CFRI Domain** | **CFIR Construct - Analytic Sub‑categories (developed after mapping)** | **Theme** |
| --- | --- | --- | --- |
| “They always say if you get any problems phone and we can probably arrange something else, but it’s like there wasn’t really any literature or whatever to continually push you forward or a website that I knew about anyway that you could access and keep going on to going forward. So that might be useful to look into. I could be wrong. There might be something you can look into, but I don’t actually remember them saying well, you can go to this website or whatever and access this and this.”  (PAT32) | **Inner Setting** | **Readiness for Implementation** 🡪 **Access to Knowledge & Information**   - No written plan - Unclear guidance about early warning signs - Inconsistent discharge information - Reliance on self‑directed problem‑solving - Feeling unsupported after treatment | Ease of and difficulties in accessing information and support🡪  Limited practical support for maintaining clinical gains after treatment  Key message: Highlights organisational gaps in the clarity, accessibility, and consistency of relapse‑prevention information, limiting patients’ ability to maintain clinical gains post‑treatment. |

Illustrative worked example of INDUCTIVE data analysis: Raw data 🡪 Indicative construct 🡪 CFIR Domain mapping 🡪 Analytic summary 🡪 Theme

| **Raw Data** | **Inductive construct** | **CFIR Domain** | **Analytic Summary** | **Theme** |
| --- | --- | --- | --- | --- |
| Being able to do what I couldn’t do before, being able to get up on a morning, and not feel like I want to sit and cry, and being able to do things that I enjoy, ‘cause I could have filled my time with all sorts of hobbies, ‘cause I do all sorts of craft hobbies, and there’s plenty of it to do in my house, but I just couldn’t even do that, ‘cause I just couldn’t be bothered… So, recovery, for me, is being able to get up, plan to do something, not cry, and just not sit and watch TV.  (PAT21) | **Meaning of recovery** | **Characteristics of Individual** | - A return to normality - Increased interest in engaging with activities and life - A key sign of progress is starting the day without overwhelming sadness or the urge to cry 🡪 gaining back control | Recovery as a personal journey which can involve a transition from apathy and disengagement to active involvement in hobbies.  Key message: Reflects recovery as a dynamic, cyclical process of self-regulation, uniquely defined by each individual’s experiences and perspectives. |
